# Protective Effects of *Olea europaea* L. Leaves and *Equisetum arvense* L. Extracts Against Testicular Toxicity Induced by Metronidazole Through Reducing Oxidative Stress and Regulating *NBN*, *INSL-3*, *STAR*, *HSD-3β*, and *CYP11A1* Signaling Pathways

**DOI:** 10.3390/toxics14010042

**Published:** 2025-12-30

**Authors:** Asmaa A. Azouz, Alaa M. Ali, Mohamed Shaalan, Maha M. Rashad, Manal R. Bakeer, Marwa Y. Issa, Sultan F. Kadasah, Abdulmajeed Fahad Alrefaei, Rehab A. Azouz

**Affiliations:** 1 Department of Pharmacology, Faculty of Veterinary Medicine, Cairo University, Giza 12211, Egypt; 2 Pathology Department, Faculty of Veterinary Medicine, Cairo University, Giza 12211, Egypt; 3 Biochemistry and Molecular Biology Department, Faculty of Veterinary Medicine, Cairo University, Giza 12211, Egypt; 4 Physiology Department, Faculty of Veterinary Medicine, Cairo University, Giza 12211, Egypt; 5 Pharmacognosy Department, Faculty of Pharmacy, Cairo University, Cairo 11562, Egypt; 6 Department of Biology, Faculty of Science, University of Bisha, Bisha 67714, Saudi Arabia; 7 Department of Biology, Jamoum University College, Umm Al Qura University, Makkah 21955, Saudi Arabia; 8 Department of Toxicology and Forensic Medicine, Faculty of Veterinary Medicine, Cairo University, Giza 12211, Egypt

**Keywords:** metronidazole, olive leaf extract, antioxidants, male infertility, horsetail extract

## Abstract

Metronidazole (MTZ), a widely used antiamoebic and antibacterial drug, has been linked to male reproductive damage. The aim of this study was to investigate *Olea europaea* L. and *Equisetum arvense* L. ethanol extracts for the protection against testicular toxicity and male infertility caused by MTZ, and to characterize the underlying mechanisms. Forty-two male rats were divided into six groups. The animals in group 1 served as the controls and received a daily oral dose (1 mL) of the vehicle. The animals in group 2 received metronidazole at doses of 400 mg/kg. Group 3 was treated with *E. arvense* extract at doses of 100 mg/kg. Group 4 was treated with *O. europaea* leaf extract at doses of 400 mg/kg. Group 5 was treated with metronidazole and *E. arvense* extract at doses of 400 and 100 mg/kg, respectively. Group 6 was treated with metronidazole with *O. europaea* leaf extract at doses of 400 and 400 mg/kg, respectively. The rats were given a daily oral dose of different treatments for 60 days, after which the animals were euthanized to study the histopathological and molecular changes in the testis and the sperm count in the epididymis. The testosterone levels, MDA levels, and GSH contents were also assessed in the rats in all groups. The findings revealed that the MTZ treatment caused a substantial increase in MDA levels and upregulated the *NBN* gene expression relative to the control. Moreover, the MTZ treatment produced significant reductions in the sperm count and viability, testosterone levels, and GSH content, and downregulated the INSL-3, STAR, HSD-3β, and CYP11A1 gene expression compared to the control. The adverse effects in testicular tissue were significantly reduced in rats given the *O. europaea* leaves and *E. arvense* treatment. The findings may show that MTZ can enhance testicular toxicity and infertility, but both plant extracts can prevent these harmful consequences.

## 1. Introduction

In recent years, male infertility has emerged as a major global health concern. Lipid peroxidation of the sperm membrane and direct damage from ROS to the nucleus and mitochondrial DNA are the primary causes of infertility in males [1]. The rise in male infertility cases brought on by the regular use of various therapeutic medications has prompted research into the negative effects of these medications on male reproduction. Several drugs have been shown to result in male infertility. Several such drugs are derivatives of nitroimidazole, including metronidazole (MTZ) [2]. Metronidazole (1-(2-hydroxyethyl)-2-methyl-5-nitroimidazole, MTZ) is used as a wide-spectrum biocide and as an antiparasitic medicine in the management of *Entamoeba hystolitca*, *Giardia lamblia*, *Trichomonas vaginalis*, and anaerobic organisms in general [3]. Its use has been limited due to the increasing evidence of its mutagenicity, carcinogenicity, and teratogenicity. MTZ causes bone marrow depression and affects male reproduction [4]. Moreover, high doses of metronidazole can cause neural and kidney damage and infertility in male rats [5].

There is worldwide interest in herbal medicinal plants [6,7]. Many people prefer herbal products to synthetic medications [8]. One of the primary benefits of using herbal products as alternative drugs is their fewer toxic side effects compared to synthetic medicines. One of the main causes of this is drug resistance, including antimicrobial drug resistance [9]. Various plants contain natural compounds that can be used to treat various medical disorders [10,11,12,13,14].

*O. europea* has long been used due to its nutritional value. In the modern era, finding a safe, natural remedy to enhance reproductive function has become challenging. Virgin olive oil affects the male reproductive system; it provides a high content of antioxidants, particularly polyphenols and other minor contents, together with an effective lipid content, characterized by the contribution of monounsaturated fatty acids [15]. The antioxidant effect of olive oil may be due to the presence of hydroxytyrosol and oleuropein as well as phenolic compounds present in this plant. Phenolics, which are naturally present in olive pomace, have a wide range of physiological properties and can decrease oxidative damage [16]. Recently, olive tree leaves have been noted for their medicinal properties and use as antioxidants, antibacterials, and anti-inflammatories [17,18]. Treatment with olive leaves has been shown to increase the quality and quantity of sperm, and enhance testosterone levels, total antioxidant capacity (TAC), and testis antioxidant status in rat testicular tissue, and the luteinizing hormone level that activates testosterone production in males [19,20,21,22].

Previous studies have demonstrated that plants in the family Equisetaceae—specifically, those in the genus *Equisetum*—are promising sources of herbal medicines. These plants can be used as alternative drugs for the treatment of numerous diseases [23,24,25]. *E. arvense*, common name horsetail, is a species in the family Equisetaceae [26,27]. *E. arvense* is rich in several chemical components, such as silicic acid, phenolics, flavonoids, glucosides, triterpenoids, saponosides, phytosterols, tannins, saponins, fatty acids, traces of alkaloids, carbohydrates, proteins, and amino acids, in addition to calcium carbonate, potassium sulfate, potassium chloride, manganese chloride, iron, manganese, and calcium phosphate [28,29]. There is an association between phenolic compounds and antioxidant effects [30]. *E. arvense* has attracted wider attention in the medical field due to its potent antioxidant capacity to ameliorate male reproductive infertility [31]. In addition, several studies have demonstrated *E. arvense*’s ability to lower oxidative-stress-related conditions [32]. In addition, studies have demonstrated various biological impacts of *E. arvense* extracts, including antibacterial, antifungal, antioxidant [33,34], anti-inflammatory [35], neuro- and cardioprotective [36,37], and antiproliferative properties [34,38]. To our knowledge, there are no reports concerning the protective effect of *O. europea* and *E. arvense* extracts against MTZ-induced testicular toxicity in rats. Hence, the aim of the present study was to investigate the potential protective role of *O. europea* and *E. arvense* leaf extracts against MTZ-induced male infertility, oxidative stress, hormonal imbalances, sperm damage, and histopathological alterations in male rats.

## 2. Materials and Methods

### 2.1. Chemicals

MTZ of analytical grade was obtained from Sigma-Aldrich (Darmstadt, Germany) (CAS number 443-48-1).

### 2.2. Plant Material

Olive leaves (*Olea europaea* L.) were harvested in October 2022 from the Experimental Station of Medicinal Plants at Cairo University’s Faculty of Pharmacy in Giza, Egypt. Concurrently, a horsetail (*Equisetum arvense* L.) specimen imported from Syria was procured from the Haraz herbal shop in Cairo. The botanical identity of both samples was authenticated by the herbarium at the Faculty of Science, Cairo University.

### 2.3. Extract Preparation

For extract preparation, the olive leaves were cleaned and dried at 40 °C, and then ground into a powder. An aqueous infusion was prepared by steeping approximately 1 kg of this powder in boiling water (10 L). The mixture was allowed to cool to room temperature before filtration, and the resulting liquid was lyophilized to yield a solid olive leaf extract. The horsetail sample was air-dried and powdered, and then extracted with ethanol. About 2 kg of the powder was repeatedly macerated in 70% ethanol (10 L × 4 times) overnight at room temperature for four days. The combined ethanol fractions were then concentrated under a vacuum at temperatures below 40 °C, producing a dark green residue. Both final extracts were stored at −40 °C for subsequent analysis and biological assays.

### 2.4. Sample Preparation for Ultra-High-Performance Liquid Chromatography–Mass Spectrometry (UPLC-ESI–QTOF-MS) Analysis

A solution was prepared by dissolving fifty milligrams of each of the ethanol extracts obtained from olive leaves and horsetail in one milliliter of reconstitution solvent consisting of a mixture of water, methanol, and acetonitrile in a volumetric ratio of 50:25:25. The resulting solutions were vortexed for two minutes, followed by ultra-sonication for ten minutes. The solutions were centrifuged at 10,000 rpm for 10 min. The stock solutions were diluted by adding 50 µL to 1000 µL of the reconstitution solvent. The concentration ultimately reached a value of 2.5 µg/µL. A volume of 10 µL from the resultant solution was introduced into the negative mode. For comparison, an equal volume of 10 µL from the solvent used for reconstitution was used as a blank sample [39].

### 2.5. High-Resolution UPLC-ESI-QTOF-MS Methodology

Chromatographic separation was achieved using a binary solvent system. Mobile phase A consisted of 5 mM ammonium formate (pH 8) with 1% (*v*/*v*) methanol, while mobile phase B was 100% acetonitrile. A linear gradient elution program was executed over 28 min, transitioning from 90% A to 90% B. An XBridge C18 column (Waters^®^, Milford, MA, USA, 50 × 2.1 mm, 3.5 µm) was used for separation at a constant temperature of 40 °C, with a mobile phase flow rate of 0.3 mL/min and a 10 µL injection volume.

### 2.6. Mass Spectrometric Conditions

A QTOF mass spectrometer was operated in negative electrospray ionization mode. The full-scan MS^1^ acquisition (TOF) covered a mass range of 50–1000 Da. The source parameters were established as follows: ion source gases 1 and 2 (GS1 and GS2) at 45, curtain gas (CUR) at 25, temperature (TEM) at 500, and ion spray voltage (ISVF) at −4500. Data-dependent MS^2^ acquisition was calibrated using an IDA method, with declustering potential (DP) set to 80, collision energy (CE) to −35, and collision energy spread (CES) to 15 [40].

### 2.7. Data Processing and Metabolite Identification

The acquired data were processed using MS-DIAL 3.52 for deconvolution and peak picking. Features were extracted by applying a signal-to-noise threshold of 5 and a minimum five-fold increase in sample intensity relative to the blanks. Putative metabolite identifications were assigned by cross-referencing experimental retention times, high-resolution accurate mass, and MS/MS fragmentation patterns with digital databases (METLIN, RIKEN) and published data. A confirmation cutoff score of 70% was applied for final assignments.

### 2.8. Animals and Experimental Design

The experimental protocol was approved by the Institutional Animal Care and Use Committee of Cairo University (CU-IACUC; Vet CU 09092023780). Forty-two male Wistar albino rats (150 ± 20 g) were acquired from the Department of Veterinary Hygiene and Management’s Animal House, Faculty of Veterinary Medicine, Cairo University. The animals were kept in standard polypropylene cages and maintained in a controlled environment with the following conditions: 55 ± 5% humidity, 22 ± 3 °C temperature, and a 12 h light/dark cycle. The rats were acclimated to the laboratory environment for 2 weeks prior to use to ensure their health. For the experimental protocol, 42 rats were randomly divided into six groups. The animals in group 1 served as the control (normal) and received a daily oral dose (1 mL) of the vehicle control. The animals in group 2 received metronidazole at doses of 400 mg/kg. Group 3 was treated with horsetail extract at doses of 100 mg/kg. Group 4 was treated with olive leaf extract at doses of 400 mg/kg. Group 5 was treated with metronidazole and horsetail extract at doses of 400 and 100 mg/kg, respectively. Group 6 was treated with metronidazole and olive leaf extract at doses of 400 and 400 mg/kg, respectively. The rats were given a daily oral dose of different treatments. The experiment lasted for 60 days.

### 2.9. Sample Collection and Preparation

At the end of the experiment, the rats were euthanized by cervical decapitation. Blood samples were collected from the retro-orbital vein and centrifuged at 4000 r/min for 10 min. Serum samples were separated for testosterone evaluation. Then, the male reproductive organs (testicles and epididymis) were excised from each rat, thoroughly washed using chilled saline (0.9% NaCl), and blotted dry. One epididymis was used for semen analysis. One testicle and the other epididymis from each rat were rapidly transferred into 10% buffered formalin for further histopathological processing. The other testicle was frozen and stored at −80 °C for subsequent molecular and oxidative stress assays.

### 2.10. Sperm Evaluation

The cauda epididymis of the testis was excised and placed in 2 mL of a 9% sodium chloride solution in a sterilized petri dish at 37 °C. Then, sterilized scissors were used to obtain a suspension of the epididymal contents. Sperm motility was evaluated using a 40× light microscope. The sperm count was estimated using a light microscope at 100×. Morphological alterations in the sperm were examined under a microscope with a magnification of 400×. About 100 spermatozoa were randomly examined under an oil-immersion objective in several fields to estimate the percentage of sperm abnormalities [41].

### 2.11. Serum Testosterone

The level of testosterone in rat serum was estimated using a competitive ELISA kit (Cat. No. 582707, Cayman Chemicals, Ann Arbor, MI, USA) following a previously described method [42].

### 2.12. Oxidative Stress

The GSH content was measured using the method described in [43]. The assay depends on the reduction of 5,5-dithiobis-2-nitrobenzoic acid by GSH, which forms a yellow product. The product’s color intensity is directly correlated with GSH levels, and the absorbance at 405 nm was measured using a spectrophotometer. MDA was measured as described in [44]. This method relies on forming a colored product by reacting MDA with thiobarbituric acid in an acidic medium. The concentration of MDA was estimated by the absorbance at a wavelength of 534 nm.

### 2.13. Real-Time Quantitative PCR Analysis of NBN, INSL-3, STAR, HSD-3β, and CYP11A1 Genes

The relative testicular mRNA expression of the *NBN*, *INSL-3*, *STAR*, *HSD-3β*, and *CYP11A1* genes was determined by quantitative real-time PCR using *ACTB* as a housekeeping gene [45]. Approximately 50 mg of testicular tissue was used for total RNA extraction with an RNA Extraction Kit (Vivantis Technologies (Biotech), Shah Alam, Malaysia). RNA concentration and purity were confirmed using a Nanodrop [46]. RT-PCR was performed using M-MuLV Reverse-Transcriptase (NEB, Ipswich, MA, USA, Cat.No.#M0253) [47]. qPCR was performed using SYBR green PCR Master Mix (Thermo Scientific, Waltham, MA, USA, Cat. No. K0221). The RT-PCR conditions were as follows: 95 °C for 5 min (initial denaturation), followed by 40 cycles at 95 °C for 15 s, 60 °C for 30 s, and 72 °C for 30 s. [48]. The primer sequences are listed in Table 1. Each qPCR was performed with three biological replicates, and each biological replicate was assessed three times [49]. Template-free negative controls were included [50]. The comparative 2^−ΔΔCT^ method was used to calculate the relative transcription levels [51].

### 2.14. Histopathological Examination

Formalin-fixed testes specimens were prepared in different grades of alcohol, cleared in xylol, and embedded in paraffin wax. Serial sections (5 μm thickness) were obtained from the prepared paraffin blocks, followed by staining with Hematoxylin and Eosin (H&E) [58].

### 2.15. Statistical Analysis

Graphs were obtained using GraphPad Prism software version 10.4.1 (GraphPad Software, San Diego, CA, USA). Data are presented as mean ± standard error (Mean ± SE). Data were analyzed using the one-way ANOVA test to estimate the difference in means. Duncan’s post hoc test was used for multiple comparisons. Statistical significance was set at *p* < 0.05 [59].

## 3. Results

### 3.1. Identification of Plant Metabolites

**Figure 1:**
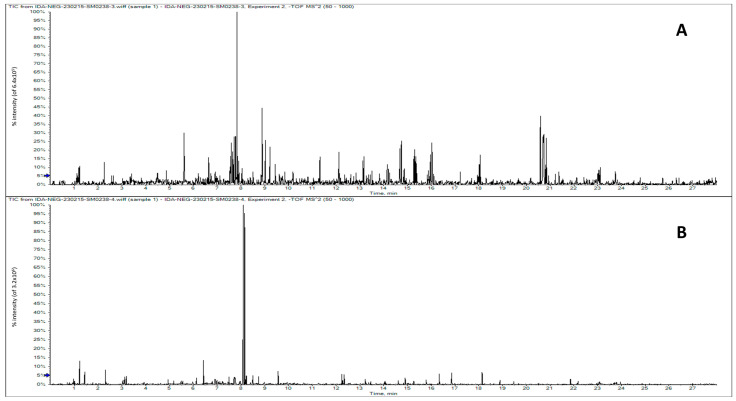
Base peak chromatograms obtained by UPLC/MS of horsetail ethanol extract (**A**) and olive leaf ethanol extract (**B**).

Table 2 provides a high-resolution comparative metabolomic profile of horsetail (*Equisetum arvense*) ethanol extract and olive leaf (*Olea europaea*) extract. The central finding is the clear chemotaxonomic distinction between the two plant extracts. Each profile is dominated by class-specific marker compounds that are well-documented, confirming their botanical origin and characteristic phytochemistry (Figure 1). The presence, absence, and distribution of compounds perfectly align with the known biosynthesis pathways of each plant family.

### 3.2. The Characteristic Metabolites of Olive Leaf Extract

The olive leaf profile was overwhelmingly defined by secoiridoids, rare iridoid monoterpenes almost exclusive to the Oleaceae family. These compounds are consistently reported as key markers [61,62,63]. The complex molecules oleuropein and ligstroside and their derivatives present in olive are responsible for the characteristic bitterness and major bioactivity of olive products [61,76]. The extract also contains loganic acid, secologanic acid, elenolic acid hexosides, and oleuropein aglycone [61,62,72]. Compounds such as verbascoside and caffeoyl secologanoside demonstrate the integration of secoiridoids with other phenolic units, a known feature of olive chemistry [61]. The simple phenol hydroxytyrosol and its glycoside are key bioactive compounds often derived from the hydrolysis of oleuropein, and their presence is a hallmark of olive material [61,62].

### 3.3. The Characteristic Profile of Horsetail Extract

In contrast, the horsetail extract lacked secoiridoids entirely. Instead, its profile was dominated by phenolic acids and a distinct pattern of flavonoids [39,60,64]. Horsetail exhibited a strong signature of caffeic acid derivatives, a characteristic of *Equisetum* species. Caffeic acid and ferulic acid, in addition to multiple caffeic acid hexosides and derivatives, were detected in horsetail extract [60,61,64,66]. Flavonoids: The flavonoid profile differs from that of the olive leaf extract. Flavonols: A high abundance of kaempferol glycosides in various forms is a key feature of the horsetail extract [64]. Flavones: Apigenin-O-hexoside was present in both extracts, but horsetail contained unique complexes, including apigenin-O-hexosyl rhamnoside [61]. Flavan-3-ols: The presence of (Epi)catechin was specific to horsetail in this assessment [39]. Other Marker Compounds: Esculin, a coumarin glycoside, is a common compound in horsetail [70]. Pinoresinol, a lignan, was detected in horsetail [65].

### 3.4. Shared Metabolites and Their Significance

The compounds common to both extracts are involved in primary metabolism or are abundant plant phenolics. Malic acid, caffeoyl-malic acid, and esculin are examples of phenolic compounds that have a broad distribution across plant families. Some flavonoids, such as luteolin-O-hexoside and apigenin-O-hexoside, are widespread in the plant kingdom and were present in both extracts. However, the diversity and abundance of their specific derivatives (e.g., more kaempferol types in horsetail) remain distinct. In conclusion, this comparative analysis demonstrates the unique phytochemical fingerprints of horsetail and olive leaf extracts. The high number of annotations with a low mass error and supporting references indicate a high-quality, reliable dataset. The data serve as a reliable tool for authenticating these two botanicals. The absence of secoiridoids in the olive leaf extract would indicate adulteration or poor quality, while their detection would confirm authenticity. The distinct compositions of the extracts explain their traditional uses. Olive leaf’s bioactivity is linked to its secoiridoid content. Horsetail’s use is associated with its phenolic acids, flavonoids, and silica.

### 3.5. Sperm Evaluation

Both the control group and the rats treated with horsetail and olive leaf extracts exhibited normal sperm motility, viability, and concentration. The group treated with MTZ showed decreases in sperm motility, viability, and concentration, in addition to an increase in sperm abnormalities compared to the control group. The simultaneous administration of both plant extracts and MTZ resulted in significant improvements in sperm motility, viability, and concentration with decreases in sperm abnormalities compared to the MTZ group (Table 3).

### 3.6. Serum Testosterone

**Figure 2:**
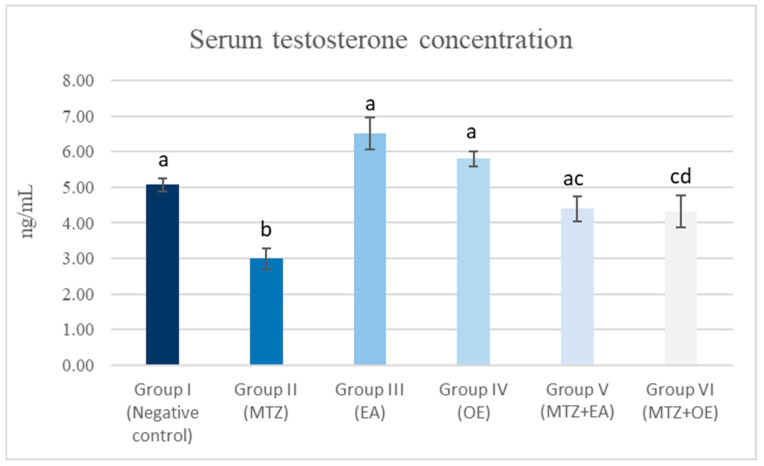
The effect *E. arvense* and *O. europaea* extracts on testosterone concentration in serum of MTZ-treated rats. Data are represented as mean ± SE. Groups having different letters are significantly different from each other at *p* < 0.05.

The MTZ group (II) exhibited a significant decrease in testosterone concentration in comparison with the control group (Figure 2; *p* < 0.05). By comparison, the EA group (III) showed a mitigation of the negative effects of MTZ, in addition to the increased production of testosterone compared to the control group (I). The OE group (IV), similar to the MTZ + EA (V) and MTZ + OE (VI) groups, exhibited the ameliorization of the MTZ-induced damage, achieving levels of testosterone similar to those of the control group.

### 3.7. Oxidative Stress

**Figure 3:**
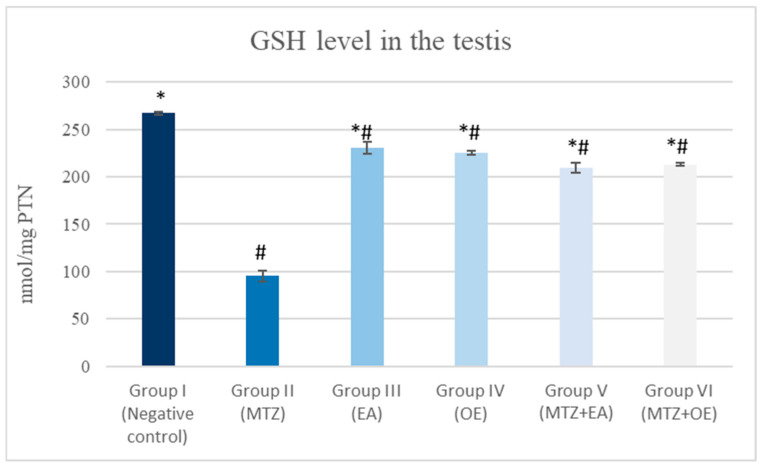
The effects of *E. arvense* and *O. europaea* extracts on GSH level in MTZ-treated rats. Data are expressed as Mean ± SD. * indicates a significant difference compared to Group I (control) at *p* < 0.05. # indicates a significant difference compared with Group II (MTZ-treated group) at *p* < 0.05.

**Figure 4:**
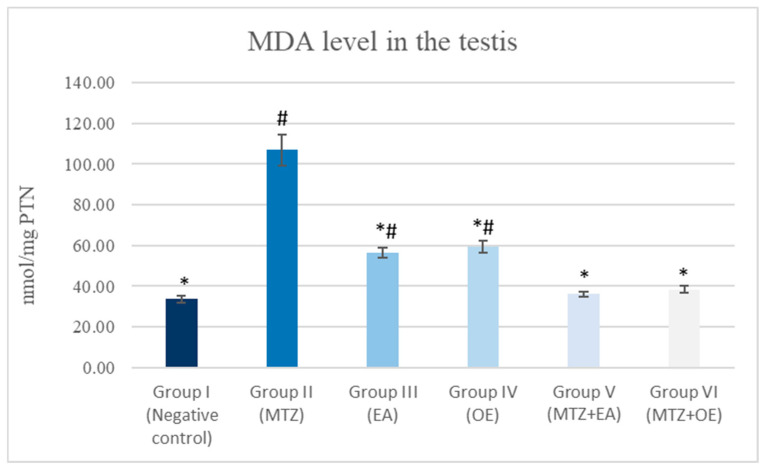
The effects of *E. arvense* and *O. europaea* extracts on MDA level in MTZ-treated rats. Data are expressed as Mean ± SD. * indicates a significant difference compared to Group II (MTZ-treated group) at *p* < 0.05. # indicates a significant difference compared to Group I (control-ve) at *p* < 0.05.

Rats treated with MTZ showed a significant decline in GSH content and an increase in MDA concentration in their testicular tissue compared to the control group. The cotreatment with *E. arvense* and *O. europaea* extracts, together with MTZ, resulted in a significant elevation in GSH levels and a lowering in MDA levels compared to the group administered only MTZ (*p* ≤ 0.05; Figure 3 and Figure 4).

### 3.8. Real-Time Quantitative PCR Analysis of NBN, INSL-3, STAR, HSD-3β, and CYP11A1 Genes

#### 3.8.1. Testicular mRNA Relative Expression of NBN Gene

**Figure 5:**
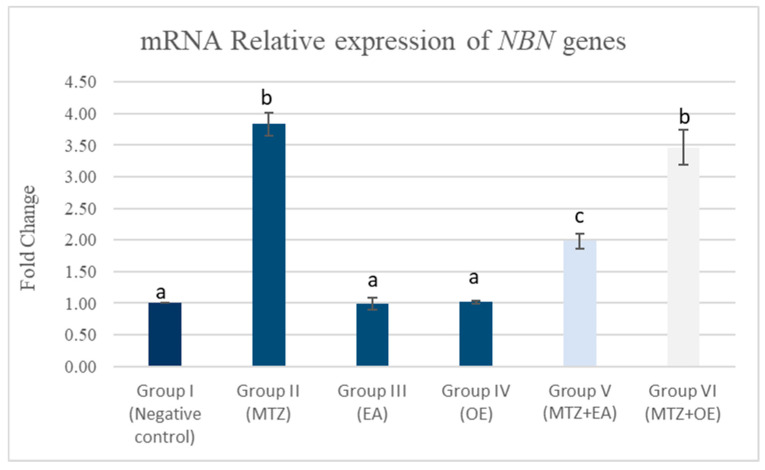
The effects of *E. arvense* and *O. europaea* extracts on *NBN* gene expression in testicular tissue of MTZ-treated rats. Data are represented as mean ± SE. Groups having different letters are significantly different from each other at *p* < 0.05.

The MTZ group (II) demonstrated a significant upregulation in the testicular mRNA relative expression of *NBN* in comparison to the control group (Figure 5; *p* < 0.05). While *E. arvense* (EA) and *O. europaea* (OE) extracts resulted in a non-significant change in the *NBN* expression in comparison to the control group, in contrast, the MTZ cotreatment with *E. arvense* alleviated MTZ-induced effects on the testicular expression of *NBN* genes compared to the MTZ group (*p* < 0.05). However, Group VI (MTZ + OE) exhibited a non-significant improvement in *NBN* expression in comparison to the MTZ group.

#### 3.8.2. Testicular mRNA Relative Expression of INSL-3 Genes

**Figure 6:**
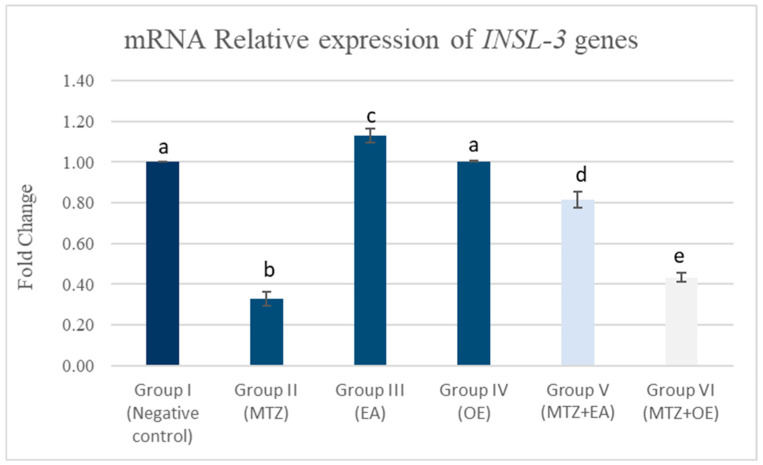
The effects of *E. arvense* and *O. europaea* extracts on INSL-3 gene expression in testicular tissue of MTZ-treated rats. Data are represented as mean ± SE. Groups having different letters are significantly different from each other at *p* < 0.05.

The MTZ group (II) showed a significant downregulation in the testicular mRNA relative expression of *INSL-3* compared to the control group (*p* < 0.05; Figure 6). Compared to the control group (I), the EA group (III) exhibited a significantly upregulated synthesis of the *INSL-3* gene, while the OE group (IV) showed a non-significant change. The MTZ cotreatment with *E. arvense* (MTZ + EA) and *O. europaea* extracts (MTZ+ OE) induced a significant upregulation of the *INSL-3* mRNA relative expression compared with the MTZ group (*p* < 0.05).

#### 3.8.3. Testicular mRNA Relative Expression of STAR, HSD-3β, and CYP11A1 Genes

**Figure 7:**
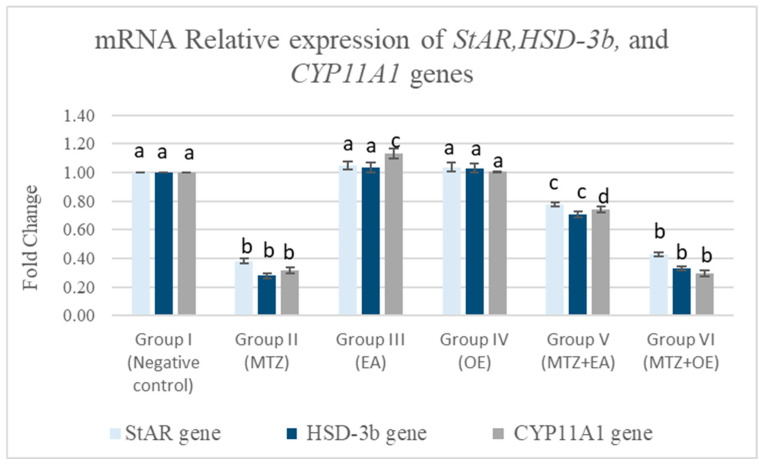
The effects of *E. arvense* and *O. europaea* extracts on *STAR*, *HSD-3β*, and *CYP11A1* gene expression in testicular tissue of MTZ-treated rats. Data are represented as mean ± SE. Groups having different letters are significantly different from each other at *p* < 0.05.

The results indicated that the MTZ treatment significantly downregulated the testicular mRNA expression of the *STAR*, *HSD-3β*, and *CYP11A1* genes in comparison with the control group (*p* < 0.05; Figure 7). Compared to the control group (I), the *STAR* and *HSD-3β* genes showed a non-significant change in expression in the *E. arvense* (EA) and *O. europaea* (OE) groups, while the EA group (III) exhibited the significantly upregulated expression of the *CYP11A1* gene. The MTZ cotreatment with *E. arvense* (MTZ + EA) significantly upregulated the testicular mRNA expression of the *STAR*, *HSD-3β*, and *CYP11A1* genes. In contrast, the *O. europaea* extract cotreatment (MTZ *+* OE) showed a non-significant change in the expression levels of the *STAR*, *HSD-3β*, and *CYP11A1* genes compared to the MTZ group.

### 3.9. Histopathology

**Figure 8:**
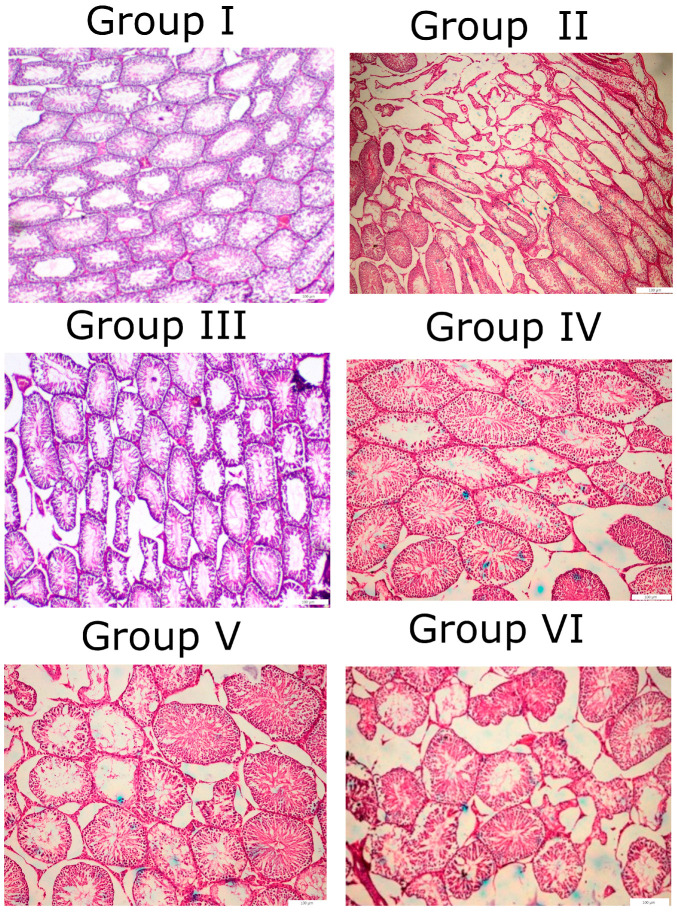
Representative histopathological images of H&E of testicular tissue. Group I: served as the control. Group II: Rats received metronidazole at doses of 400 mg/kg. Group III: Rats were treated with horsetail extract at doses of 100 mg/kg. Group IV: rats were treated with olive leave extract at doses of 400 mg/kg. Group V: rats were treated with metronidazole and horsetail extract. Group VI: Rats were treated with metronidazole and olive leaf extract.

The control rats had a normal histological structure, characterized by densely packed seminiferous tubules and a synchronized population of mature germ cells (Figure 8). Group II displayed significant histological abnormalities, including severe germ cell degeneration and mortality, along with the minor vacuolation of Sertoli cells. The atrophy of seminiferous tubules accompanied by a reduction in Leydig cell quantity was prevalent in this cohort. Nonetheless, there was no substantial difference between Group III and Group IV compared to Group I. Group V exhibited the degeneration and death of certain germ cells in seminiferous tubules, but to a lesser extent than in Group II. Conversely, Group VI exhibited significant germ cell degradation and mortality, comparable to that of Group II. Ultimately, we concluded that metronidazole and horsetail extract, administered at respective doses of 400 and 100 mg/kg, significantly improved the histological condition of the testicular tissues.

## 4. Discussion

Most chemical compounds have not been examined for their toxic effects on sperm function, due to the fact that drug testing is mainly applied to general animal health. Antimicrobial drugs present in semen without harming sperm are essential in animal artificial insemination programs. Metronidazole is a highly effective drug widely used for the treatment of vaginal pathogens, where sperm can be directly exposed [78]. The adverse effects of MTZ on sperm have not been evaluated. Therefore, the effects of metronidazole on rat sperm viability and male reproductive ability were examined in the present study.

Exposure to metronidazole resulted in a persistent lowering of testosterone levels in rats. The decline in the weight of the testes, epididymides, and accessory sexual organs recorded in this study may have been due to a decrease in the concentration of testosterone. The administration of metronidazole for one month decreased the levels of testosterone, FSH, and LH in male rats [79]. In the present study, metronidazole caused a significant reduction in the percentage of motile sperm, while sperm cell abnormalities were significantly increased. Moreover, a single dose of 2-thiazolyl-5-nitroimidazole resulted in infertility in mice after three weeks [80]. The decrease in the percentage of motile sperm and the increase in abnormal sperm may have been due to metronidazole, as it can penetrate the blood–testis barrier and affect the germ cells in the seminiferous tubules. The blood–testis barrier is an important consideration when assessing the reproductive and mutagenic effects of medicines and pollutants [81]. Metronidazole reaches all tissues, including the brain and seminal fluid [82]. Metronidazole immobilized rat sperm in vitro [83]. Metronidazole affected the percentages of motile rabbit and human spermatozoa [78]. The results of these studies demonstrate the direct toxic effects of metronidazole on sperm and Leydig cells; i.e., it reduced testosterone production after the penetration of the blood–testes barrier.

Environmental toxins are considered contributors to male fertility [84,85]. Factors such as oxidative stress, systemic inflammation, excessive ejaculation frequency, and obesity can lead to a decline in semen quality [86]. Research findings indicate that elevated levels of ROS in the testes of aged mice have led to a reduction in steroidogenic enzymes, resulting in decreased levels of steroid hormones [87]. The results of our study show a significant increase in MDA levels, a biomarker of lipid peroxidation, and a substantial decrease in reduced glutathione content, a biomarker of antioxidant activity, in animals treated with MTZ compared to the control group. Oxidative stress is enhanced by various environmental pollutants, the presence of infection, and the metabolic processing of ingested food [88]. Studies have shown that damage to sperm by ROS is a significant contributing histopathology, comprising 30–80% of cases [89]. In particular, the peroxidation of the sperm phospholipids not only reduces membrane flexibility and tail motion [90] but also affects mitochondrial function, further impairing energy generation [91]. More ROS inside cells affects sperm motility, thereby impairing penetration and fertilization [92]. In addition, when the sperm are affected by oxidative stress, natural conception will prevent abnormal sperm from producing embryos [93].

An imbalance between oxidant and antioxidant agents results in the release of free radicals and reactive oxygen species (ROS), which, in turn, leads to oxidative stress [94]. Horsetail and olive leaf extracts attenuated the MDA concentration induced by MTZ treatment. MDA levels are proportional to lipid peroxidation and are markers of oxidative damage [95,96]. MDA is considered a degradation product of lipids resulting from ROS attacking polyunsaturated fatty acids, and it is associated with cellular toxicity or stress [97,98]. The decline in GSH content in MTZ-exposed rats was subsequently mitigated by the administration of both plant extracts. Lower GSH levels are considered an important biomarker of oxidative damage due to their greater utilization by tissues under stress [95]. The findings indicate the possible restorative ability of both plant extracts against MTZ-induced oxidative damage. The co-administration of both plant extracts significantly enhanced the GSH content in testicular tissue and reduced oxidative stress by decreasing lipid damage and inhibiting ROS production.

Olive leaf extract contains various polyphenolic compounds, flavonoids, and secoiridoids [99]. Among phenolic compounds, oleuropein, luteolin, and hydroxytyrosol exhibit strong antioxidant activity [100]. The current experimental findings showed that the administration of olive leaf extract for 60 days significantly improved the sperm quality and antioxidant status in the testis of rats exposed to MTZ. The administration of 300 mg/kg OLE markedly reduced the testis MDA concentration and enhanced the sperm quality [19]. Similarly, oleuropein ameliorated the impacts of alcohol-induced oxidative stress in male rat testes and alleviated the damage to sperm function [101]. In group 6 (metronidazole with olive leaf extract), we observed a lower semen quality compared to the untreated control group. This may be explained by the dosage used and the duration of treatment. In agreement with this result, another study found that the administration of olive fruit extract had negative impacts on sperm parameters [102]. *E. arvense* contains numerous promising bioactive constituents belonging to different chemical classes in addition to minerals [28,29]. Due to its chemical composition, *E. arvense* possesses a powerful antioxidant capacity, enabling it to prevent male infertility [103]. Moreover, several studies have reported the potential of horsetail extract in lowering oxidative-damage-related conditions [104,105]. Based on the above findings, we conclude that MTZ induces testicular damage by imbalancing the testicular antioxidant status.

Our study investigated the histopathological effects of horsetail extract (*Equisetum arvense*) and olive leaf extract (*Olea europaea*) in ameliorating the effects of metronidazole on rat testes. As expected, the control group (Group I) displayed normal histological features, including well-organized seminiferous tubules and synchronized germ-cell maturation. In contrast, Group II (metronidazole, 400 mg/kg) exhibited severe testicular pathology, including germ-cell degeneration, Sertoli cell vacuolation, tubular atrophy, and a marked reduction in Leydig cell numbers. These findings are consistent with earlier reports that high-dose metronidazole disrupts spermatogenesis and induces infertility through germ-cell apoptosis, seminiferous epithelial degeneration, and hormonal imbalance [106,107]. The groups treated with horsetail extract (Group III, 100 mg/kg) or olive leaf extract (Group IV, 400 mg/kg) showed no significant deviations from the control group in histology, suggesting that both extracts are well-tolerated and may have protective effects on the testicular structure. Horsetail extract is known for its antioxidant and anti-inflammatory activities, which have been reported to protect reproductive tissues from toxic insults [103]. Similarly, olive leaf extract is rich in polyphenols, particularly oleuropein, which possesses strong free-radical scavenging activity and has been shown to mitigate testicular oxidative damage in models of diabetes and drug toxicity [22,108].

The combined treatment groups further support this protective role. Group V (metronidazole + horsetail) displayed reduced germ-cell degeneration compared to the metronidazole-only group, highlighting the ameliorative effect of horsetail extract. This finding may be attributed to its ability to restore antioxidant enzyme activity and reduce lipid peroxidation, as observed in other models of chemically induced testicular injury [109]. Conversely, Group VI (metronidazole + olive leaf extract) still demonstrated significant germ-cell mortality, comparable to Group II. This suggests that, while olive leaf extract possesses protective properties, its efficacy may be dose-dependent and thus may have been insufficient at the tested concentration to fully counteract metronidazole toxicity. Our findings demonstrate that 100 mg/kg horsetail extract exhibited a stronger protective influence on testicular histology compared to olive leaf extract at 400 mg/kg. The superior effect of horsetail may be linked to its higher flavonoid and silica content [110,111], which enhances its antioxidant capacity and supports germinal epithelium recovery.

MTZ is well-established as a potent inhibitor of steroidogenesis and spermatogenesis [112,113,114,115], but the exact molecular mechanism remains unknown. Thus, our current study aimed to determine the possible molecular mechanisms underlying MTZ-induced effects and the possible ameliorative effects of *Equisetum arvense* L. Leydig cells are essential for steroidogenesis and spermatogenesis. Oxidative stress results in Leydig cell dysfunction, resulting in the impairment of steroidogenesis and decreased testosterone concentration, spermatogenesis, and, ultimately, male infertility [116]. The process of steroidogenesis is initiated with the conversion of cholesterol to pregnenolone within the mitochondria by cholesterol side-chain cleavage by the enzyme cytochrome P450 (*CYP11A1*). Cholesterol transport within the mitochondria is facilitated by *StAR*. Pregnenolone is then transformed into other steroids by a series of oxidative enzymes located in both the mitochondria and endoplasmic reticulum [117]. Our current results revealed that the oral administration of MTZ significantly downregulated several testicular-steroidogenesis-related genes (*StAR*, *HSD3β*, and *CYP11A1*), suggesting a potential role in inhibiting steroidogenesis. *StAR* regulates the delivery of cholesterol from the outer mitochondrial membrane to the inner mitochondrial membrane, the rate-limiting step in steroid biosynthesis, and thus plays an essential role in regulating steroidogenesis [118]. *CYP11A1* is a protein-coding gene that catalyzes the conversion of cholesterol to pregnenolone, the precursor of most steroid hormones [119]. *HSD-3β* is a protein-coding gene catalyzing the conversion of 3β-hydroxysteroids into 3-keto-steroids, which leads to the synthesis of all classes of steroid hormones [120]. In the current study, the inhibition of Leydig cell steroidogenesis was associated with significant oxidative stress in testicular tissue, as confirmed by alterations in oxidative stress biomarkers in the testicular homogenate and the upregulation of *NBN* and the downregulation of *INSL3* gene expression. This result was supported by the finding that the infertility side effect of MTZ was associated with significant oxidative stress in testicular tissue homogenates [54]. In agreement with our results, MTZ administration (15, 200, or 400 mg/kg) for eight weeks induced testicular oxidative stress in male rats [121]. Additionally, the present results were similar to those of a study [122] demonstrating that the oral administration of MTZ at a dose of 500 mg/kg for 28 days was associated with oxidative stress in testicular homogenates in male Swiss mice. The *NBN* gene encodes the nibrin protein, a DNA damage response gene involved in numerous basic cellular functions, including the DNA repair of damaged lesions. *NBN* production is stimulated by DNA damage to aid in double-strand repair and genomic stability [123]. *INSL3* is a constitutive hormone secreted exclusively by Leydig cells in a gonadotropin-independent manner. It is considered a reliable biomarker of Leydig cell function [124]. Therefore, a reduced *INSL3* expression is primarily associated with dysfunction and damage to Leydig cells [53]. Taken together, the abovementioned results indicated that the MTZ-induced downregulation of steroidogenesis enzymes is related to oxidative-stress-mediated DNA damage of Leydig cells, which resulted in its dysfunction.

Our current results revealed that oxidative-stress-induced steroidogenesis disorder was abolished by co-treatment with *Equisetum arvense* L., more than *Olea europaea* L. *Equisetum arvense* possesses a potent antioxidant capacity that enables it to improve Leydig cell oxidative damage and functionality [31,103]. Several previous reports confirmed that *Equisetum arvense* extract contains high amounts of total antioxidants, total phenols, and total flavonoids [125,126,127], and other previous studies obtained similar active constituents of *Equisetum arvense* L. using GC/MS [36,128].

## 5. Conclusions

This study demonstrates that MTZ induces significant testicular toxicity characterized by oxidative stress, histopathological alterations, and DNA damage. However, the co-administration of *E. arvense* and *O. europaea* extracts effectively ameliorated these deleterious effects, likely due to their potent antioxidant properties. Both extracts restored the antioxidant status by increasing GSH levels and reducing MDA levels, thereby preserving sperm viability and testicular histology. *E. arvense* exhibited a superior protective efficacy compared to *O. europaea*, particularly in restoring the expression of steroidogenesis-related genes and mitigating germ cell degeneration. These findings suggest that *E. arvense* is a promising therapeutic agent for preventing drug-induced male infertility.

## Figures and Tables

**Table 1:** Primers used for real-time PCR.

Gene Symbol	Gene Description	Accession Number	Primer Sequence
*NBN* [52]	Nibrin	NM_138873.2	F: 5′-CTTCAGGACAGCAGTGAGGA-3′ R: 5′-TCTTTCGAGCATGGTGACCT-3′
*INSL-3* [53]	Insulin-like growth factor -3	NM_053680	F: 5′-GTGGCTGGAGCAACGACA-3′ R: 5′-AGAAGCCTGGTGAGGAAGC-3′
*STAR* [54]	Steroidogenic Acute Regulatory	NM_031558.3	F: 5′-GCC TGA GCA AAG CGG TGT C-3′ R: 5′-CTG GCG AAC TCT ATC TGG GTCTGT-3′
*HSD-3β* [55]	Hydroxy-Delta5-Steroid Dehydrogenase, 3 Beta- And Steroid Delta Isomerase 1	NM_001007719.3	F: 5′-CTCACATGTCCTACCCAGGC-3′ R: 5′-TATTTTTGAGGGCCGCAAGT-3′
*CYP11A1* [56,57]	Cytochrome P450 11A1	NM_017286.3	F: 5′-GCT GGAAGG TGT AGC TCA GG-3′ R: 5′-CAC TGG TGT GGA ACA TCT GG-3′
*ACTB* [45]	Actin beta	NM_031144.3	F: 5′-TGTCACCAACTGGGACGAT-3′ R: 5′-GGGGTGTTGAAGGTCTCAA-3′

**Table 2:** Identification of phytochemical compounds in *Olea europaea* L. leaves and *Equisetum arvense* L. ethanol extract by UPLC-ESI–QTOF-MS Analysis.

Rt	M-H	Error	Name	Molecular Formula	Fragments	Horsetail Ethanol Extract	Olive Leaf Ethanol Extract	Reference	Class
1.05	133.0142	0.00	Malic acid	C4H6O5	115,89,73,71	+	+	[60]	Organic acid
1.07	173.0455	0.00	Shikimic acid	C7H10O5	155,137,129,111,93,85	+	−	[60]	Phenolic acid
1.09	317.0305	0.66	Myricetin	C15H10O8	299,225,165, 81	−	+	[61]	Flavonoids
1.10	195.0511	0.38	Gluconic acid	C6H12O7	177,159,129,99,87,75	−	+	[62]	Organic acid
1.11	165.0404	−0.38	Pentose acid	C5H10O6	147,129,105,87	−	+	[63]	Saccharide
1.11	179.0352	1.22	Caffeic acid	C9H8O4	135,113,101,89,71,59	+	−	[60]	Phenolic acid
1.11	191.0560	−0.59	Quinic acid	C7H12O6	173,127,109,93,85	−	+	[61]	Cyclitol
1.12	295.0460	0.20	Caffeoyl-malic acid	C13H12O8	193,179,151,133,115	+	+	[64]	Phenolic acids
1.16	187.0975	−0.44	Azelaic acid	C9H16O4	---	+	−	[65]	Organic acid
1.17	279.0539	10.30	Malic acid p-coumarate	C13H12O7	163,99	+	−	[64]	Phenolic acids
1.18	509.1662	−0.49	Demethyl ligstroside	C24H30O12	463,421,347,233	−	+	[61]	Secoiridoids
1.19	377.0855	−6.12	Caffeic acid derivative	C18H18O9	341,215,195,179,153,129	+	+	[66]	Phenolic acids
1.21	305.0698	10.24	(Epi)gallocatechin	C15H14O7	225,97,59	−	+	[67]	Flavonoids
1.21	343.1029	−1.62	Hydrocaffeic acid hexoside	C15H20O9	181,179,161,119	+	+	[64]	Phenolic acids
1.21	389.1090	0.17	Oleoside/secologanoside	C16H22O12	345,209,183,165,121,89,69	−	+	[61,62]	Secoiridoids
1.22	215.0328	−0.85	Bergapten	C12H8O4	179,161,149,113,89,71	+	−	[68]	Furanocoumarins
1.23	181.0716	−0.89	Sugar alcohol	C6H14O6	163,149,119,101,89,71,59	+	−	[63]	Saccharides
1.24	179.0560	−0.63	Hexose	C6H12O6	135,89,71,59	+	+	[63]	Saccharides
1.26	341.1089	3.50	Caffeic acid hexoside	C15H18O9	179,161,119,89,59	+	+	[61]	Phenolic acids
1.26	387.1144		Caffeic acid derivative	---	341,179,161,119,89	+	+	[66]	Phenolic acids
1.26	731.2211	2.58	Oleoside/secologanoside derivative	C35H40O17	389,343,227	−	+	[69]	Secoiridoids
1.28	181.0718	0.21	Sugar alcohol isomer	C6H14O6	163,149,119,101,89,71,59	+	−	[63]	Saccharides
1.28	375.1294	−0.72	Loganic acid	C16H24O10	331,317,213,195,165	−	+	[62]	Secoiridoids
1.28	401.1238	−0.97	Hexamethoxyflavone	C21H22O8	355,341,193,179,161	+	−		Flavonoids
1.28	407.1552	−1.68	Acyclodihydroelenolic acid hexoside	C17H28O11	389,377,363,345,233,151	−	+	[61]	Secoiridoids
1.28	447.1140	−0.30	Dihyroxybenzoic acid hexoside pentoside	C18H24O13	349,265,195,152	−	+	[65]	Phenolics
1.28	537.1825	−0.98	Loganic acid hexoside	C22H34O15	537,375	−	+	[67]	Secoiridoids
1.28	731.2236	5.92	Oleoside/secologanoside derivative	C35H40O17	389,345,343,227	−	+	[69]	Secoiridoids
1.30	685.2342	−1.06	(Iso)Nuzhenide	C31H42O17	523,453,421,403,387	−	+	[61]	Secoiridoids
1.39	213.0769	0.25	Decarboxy-hydroxy-elenolic acid	C10H14O5	183,169,151,139,107	−	+	[63]	Phenol ethers
1.76	151.0400	−0.45	Oxidized hydroxytyrosol	C8H8O3	123,109,95,81,77	−	+	[61]	Phenylalcohols & derivatives
1.99	403.1242	−0.96	Elenolic acid hexoside/Oleoside methylester	C17H24O11	371,333,223,179,119,89	−	+	[61]	Secoiridoids
2.02	197.0820	0.35	Decarboxymethyl elenolic acid dialdehyde	C10H14O4	153,137,123	−	+	[63]	Secoiridoids
2.22	435.1295	−0.39	Phlorizin	C21H24O10	273,255,229,123	+	−	[64]	Others
2.35	315.1086	0.19	Hydroxytyrosol hexoside	C14H20O8	153,135,123	−	+	[62]	Secoiridoids
2.38	403.1969	−1.13	2-(2-ethyl-3-hydroxy-6-propionylcyclohexyl) acetic acid glucoside	C19H32O9	223	−	+	[65]	Others
2.39	339.0720	−0.46	Esculin	C15H16O9	177,133	+	+	[70]	Coumarins
2.41	353.0876	−0.58	Caffeoylquinic acid	C16H18O9	315,191,173,153,124	−	+	[61]	Phenolic acids
2.57	193.0507	0.35	Ferulic acid	C10H10O4	149,121,77	+	−	[60]	Phenolic acid
2.64	313.0927	−0.61	Vanillin hexoside	C14H18O8	151,123	−	+	[61]	Phenolic aldehyde
3.19	153.0558	0.00	Hydroxytyrosol	C8H10O3	123,95,77	−	+	[61]	Phenylalcohols & derivatives
3.29	551.1408	0.31	Caffeoyl secologanoside (Cafselogoside)	C25H28O14	507,389,341,281,251,161	−	+	[61]	Secoiridoids
3.94	525.1601	−2.28	Demethyl-oleuropein	C24H30O13	389,363,319,249,209,165	−	+	[61]	secoiridoids
4.19	601.1534	−4.79	Elenolic acid glucoside derivative	C29H29O15	445, 403,223,179	−	+	[63]	Secoiridoids
4.20	565.1773	−0.19	Elenolic acid dihexoside	C23H34O16	505,445,403,371,265,223,179, 89	−	+	[61]	Secoiridoids
4.44	625.1409	−0.19	Quercetin-O-dihexoside	C27H30O17	463,301	+	−	[71]	Flavonoids
4.46	289.0717	−0.21	(Epi)catechin	C15H14O6	245,203	+	−	[39]	Flavonoids
4.49	489.1605	−1.77	Caffeic acid dihexoside	C21H30O13	265,163,145	−	+	[67]	Phenolic acid
4.59	403.1230	−1.48	Elenolic acid hexoside/Oleoside methylester	C18H26O13	371,269,223,179,89	−	+	[61]	Secoiridoids
4.80	755.2042	0.24	Kaempferol coumaroyl diglucoside	C33H40O20	593,446,285	+	−	[64]	Flavonoids
4.83	609.1456	−0.83	Kaempferol-di-O-hexoside	C27H30O16	489,447,327,285	+	−	[64]	Flavonoids
4.96	401.1448	−1.30	Secologanic acid	C18H26O10	269,233,161,101	−	+	[72]	Irridoid precursor
4.97	335.1136	0.00	Hydroxy-oleacin	C17H20O7	199,181,155,111,85	−	+	[61]	Secoiridoids
5.15	389.1446	−1.85	Loganin	C17H26O10	357,313,253,151,101	−	+	[62]	Secoiridoids
5.29	385.1864	−1.02	Icariside B2	C19H30O8	223,205,153	+	+	[73]	Flavonoids
5.29	431.1918 (385 + 46)	3.84	Sinapoyl-hexoside	C17H22O10	385,223,205,161,153	+	+	[64]	Hydroxycinnamic acids
5.36	609.1456	−0.83	Quercetin hexoside deoxyhexoside	C27H30O16	447,301	−	+	[64]	Flavonoids
5.48	361.0927	>10	Ligstrosideaglycone	C19H22O7	317,225,165,137,95	−	+	[63]	Secoiridoids
5.48	535.1471	2.59	Comselogoside	C25H28O13	489,389,265,205	−	+	[65]	Secoiridoids
5.50	349.1289	−1.08	Decarboxy-hydroxy-elenolic acid linked to hydroxytyrosol	C18H22O7	331,300,271,213,181,111	−	+	[63]	Secoiridoids
5.50	687.2131	−1.58	Demethyl-oleuropein hexoside	C30H40O18	525,389,319,195	−	+	[61]	Secoiridoids
5.62	651.1563	−0.57	Quercetin-acetyl-di-hexoside	C29H32O17	531,489, 446,327,285	+	−	[64]	Flavonoids
5.64	391.1403	1.17	Methyl oleuropein aglycone	C20H24O8	345,265,235,229,193,134	−	+	[62]	Secoiridoids
5.77	423.0923	−2.33	Maclurin-O-hexoside	C19H20O11	287,261,219	+	−	[64]	Benzophenones
5.89	739.2440	−2.01	Oleuropein derivative	C34H44O18	701,539,377,307	−	+		Secoiridoids
5.95	415.1602	−1.86	Phenylethyl primeveroside	C19H28O10	149,251,221,191,89	−	+	[61]	Simple phenols
5.99	739.2091	0.00	Kaempferol-O-deoxyhexose-O-hexose-deoxyhexoside	C33H40O19	593,431,285	+	−	[64]	Flavonoids
6.00	393.1193	0.49	Hydroxy oleuropein aglycone	C19H22O9	361,345,317,289,257,181,137	−	+	[61]	Secoiridoids
6.00	491.0830	−0.23	Isorhamnetin-O-glucuronide	C22H20O13	315,300	+	+	[63]	Flavonoids
6.01	741.2488	3.94	Oleoside dimethylester diglucoside	C30H46O22	579, 417	−	+	[72]	Secoiridoids
6.10	481.1917	−2.01	Hydroxytyrosol rhamnoside	C20H34O13	417,213,153	−	+	[70]	Hydroxycinnamic acids
6.14	303.0509	−0.42	Taxifolin	C15H12O7	285,259,177,151,125	−	+	[67]	Flavonoids
6.16	583.2014	−3.14	Lucidumoside C	C27H36O14	537,461,389,375,313	−	+	[61]	Secoiridoids
6.46	555.1711	−1.49	Hydroxy-oleuropein/Secologanoside	C25H32O14	537,403,393,323,291,223,151	−	+	[61]	Secoiridoids
6.49	593.1497	−2.52	Kaempferol hexoside deoxyhexoside	C27H30O15	447,285	+	+	[64]	Flavonoids
6.67	557.2248	1.50	Oleoside-O-(hydroxy-dimethyl-octenoyl)	C26H38O13	539,511,405,395,343,325,227,185,151	−	+	[66]	Secoiridoids
6.68	449.1071	−4.09	Eriodictyol-O-hexoside	C21H22O11	287	+	+	[39]	Flavonoids
6.72	375.1429	−5.40	Olivil	C20H23O7	327,195,179	−	+	[62]	Secoiridoids
6.75	421.1702	−3.17	Dihydro Oleoside dimethylester	C18H30O11	359,239,165,119	−	+	[66]	Secoiridoids
6.84	635.1613	−0.72	Kaempferol-O-(acetyl-hexoside)-O-deoxyhexoside	C29H32O16	489,431,285	+	−	[64]	Flavonoids
6.89	447.0927	−1.31	Luteolin-O-hexoside	C21H20O11	285,255,227,151	+	+	[61]	Flavonoids
6.92	287.0557	−1.43	Eriodictyol	C15H12O6	259,243,177,151,125	−	+	[74]	Flavonoids
7.03	311.0402	−2.11	Caftaric acid	C13H12O9	243,179,161,135	−	+	[39]	Phenolic acid
7.06	327.2174	−0.91	Trihydroxy octadecadienoic acid	C18H32O5	283,229,211,171	+	+	[63]	Fatty acids
7.08	623.1972	−1.51	Verbascoside	C29H36O15	461,161	+	+	[61]	Secoiridoids
7.10	543.2076	−1.31	Dihydro oleuropein	C25H36O13	525,513,407,389,377,357,313,197,151,119	−	+	[70]	Secoiridoids
7.17	701.2292	−0.91	Oleuropein hexoside	C31H42O18	539,437,377,307,275,223,179,149,89	−	+	[61]	Secoiridoids
7.17	723.2123	−2.61	Ligustroflavone	C33H40O18	561,543,491,459,437,329,297	−	+	[71]	Flavonoids
7.25	463.0876	−1.26	Myrecetin-O-deoxyhexoside	C21H20O12	316,301,287,271,257	+	−	[64]	Flavonoids
7.25	535.1813	−1.49	Hydroxypinoresinol -O-hexoside	C26H32O12	355,295,179	−	+	[67]	Secoiridoids
7.28	419.1344	−0.85	Deoxyphlorizin	C21H24O9	257,239	+	−	[64]	Others
7.33	477.1399	−0.70	Calceolarioside A	C23H26O11	323,314,161	−	+	[65]	Pentacyclic triterpenes
7.40	447.0918	−3.32	Luteolin-O-hexoside isomer	C21H20O11	285,347	−	+	[61]	Flavonoids
7.40	607.1639	−4.85	Diosmin	C28H32O15	461,299,145	−	+	[67]	Flavonoids
7.49	431.0978	0.95	Apigenin-O-hexoside	C21H20O10	269,268	+	+	[61]	Flavonoids
7.52	539.2127		*threo*-7,9,9′-Trihydroxy-3,3′-dimethoxy-8-*O*-4′-neolignan-4-*O*-*β*-D-glucopyranoside	C26H36O12	493,361	+	−	[73]	Lignans
7.53	593.1501	−1.84	Luteolin-O-rutinoside	C27H30O15	447,285	−	+	[63]	Flavonoids
7.56	433.1131	−2.12	Naringenin hexoside	C21H22O10	271	+	−	[75]	Flavonoids
7.57	505.0945	−8.44	Trihydroxy dimethoxyflavone glucuronide	C23H22O13	341,329,300,271	+	−	[64]	Flavonoids
7.61	325.0928	−0.28	Coumaroylhexose	C15H18O8	163	+	−	[66]	Phenolics
7.61	329.1392		Trihydroxy dimethoxyflavone	C17H14O7	314,299,269,229,211	+	−	[60]	Flavonoids
7.62	671.2190	−0.41	Oleuropein pentoside	C30H40O17	539,377,307,275,149	−	+	[71]	Secoiridoids
7.64	195.0660	−1.03	Hydroxytyrosol acetate	C10H12O4	177,159,151,135,107	−	+	[70]	Phenylalcohols & derivatives
7.68	329.2329	−1.36	Trihydroxy octadecenoic acid	C18H34O5	314,299,269,229,211,193,171,139	+	+	[63]	Fatty acids
7.69	463.0881	−0.21	Quercetin-O-hexoside	C21H20O12	301	−	+	[67]	Flavonoids
7.71	461.1084	−1.16	Diosmetin-O-hexoside	C22H22O11	299,285,255,145	−	+	[67]	Flavonoids
7.72	287.2226	−0.63	Dihydroxypalmitic acid	C16H32O4	269	+	+	[66]	Fatty acids
7.81	593.1534	3.72	Vicenin 2	C27H30O15	547,473,383,353,325,297	−	+	[69]	Flavonoids
7.83	489.1034	−0.92	Kaempferol-O-acetyl-hexoside	C23H22O12	327,284,285,255,227	+	−	[39]	Flavonoids
7.88	377.1238	−1.04	Eriodictyol derivative	C19H22O8	287,269,257,229,163	+	−	[39]	Flavonoid
7.89	541.1911	−2.89	Hydro oleuropein	C25H34O13	361,329,225,193,181,149,121,89	−	+	[70]	Secoiridoids
7.93	577.1608	7.83	Apigenin-O-hexosyl rhamnoside	C27H30O14	415,269	−	+	[70]	Flavonoids
8.15	579.1692	−4.71	Naringin	C27H32O14	543,525,513,389,377,271	−	+	[39]	Flavonoids
8.17	539.1762	−0.77	Oleuropein	C25H32O13	539,377,307,275,223,179,149,89	−	+	[76]	Secoiridoids
8.24	597.1349		Oleuropein derivative		539,507,377,307,275,223,149	−	+	[69]	Secoiridoids
8.43	753.2250	0.33	Trihydroxy-methoxyflavone-O-deoxyhexose-deoxhexose-hexoside	C34H42O19	299	+	−	[64]	Flavonoids
8.51	307.1915		Catechin hydrate	C15H16O7	289,235,211,185,169,121,97	+	−	[39]	Flavonoids
8.85	537.1601	−2.35	Fraxamoside	C25H30O13	403,223,151	−	+	[61]	Secoiridoids
9.00	523.1812	−1.72	Ligstroside	C25H32O12	361,291,259	−	+	[61]	Secoiridoids
9.03	293.1756	−0.79	Gingerol	C17H26O4	236,221,205,177	−	+	[65]	Phenolic
9.12	925.2980	−0.33	Jaspolyoside	C42H54O23	749,701,539,377,307,149	−	+	[71]	Flavonoids
9.21	847.2677	1.28	Oleuropein derivative	C40H48O20	685, 583,539, 377,307	−	+	[69]	Secoiridoids
9.36	877.2771	−0.09	Oleuropein derivative	C41H50O21	715,539	−	+	[69]	Secoiridoids
9.94	377.1237	−1.30	Oleuropeine aglycone	C19H22O8	241,327,307,199,153	−	+	[61]	Secoiridoids
10.81	357.1338	−1.57	Pinoresinol	C20H22O6	295, 221,189,122,83	+	−	[65]	Lignan
11.29	375.1079	−1.70	Dehyro-Oleuropein aglycone	C19H20O8	375,343,207,195,189, 177,163,135	−	+	[61]	Secoiridoids
11.34	285.0402	−0.70	Kampferol (tetrahydroxyflavone)	C15H10O6	257,239,229,185,151,131	+	−	[74]	Flavonoids
12.19	373.1287	−1.55	Hydroxy pinoresinol/Africanal	C20H22O7	311,289,237,163,119	−	+	[66]	Lignan
12.29	315.0480	−9.60	(Iso)rhamnetin	C16H12O7	300,163,145	−	+	[61]	Flavonoids
12.32	293.2120	−0.74	Hydroxy-octadecatrienoic acid	C18H30O3	275,235,223,183,171	+	−	[39]	Fatty acids
12.68	271.2277	−0.62	Hydroxyhexadecanoic acid	C16H32O3	---	+	−	[39]	Fatty acids
12.76	313.0712	−1.80	Cirsimaritin	C17H14O6	298,283,253,225, 197,163,143,119	+	−	https://pubchem.ncbi.nlm.nih.gov/compound/Cirsimaritin#section = MS-MS (accessed on 1 September 2025)	Flavonoids
13.47	345.1705	−0.72	Epi(rosmanol)	C20H26O5	301,283,257,177,133	+	−	[60]	Phenolic terpenes
13.61	295.2274	−1.59	Hydroxy-octadecadienoic acid	C18H32O3	277,195,171	+	−	[39]	Fatty acids
17.87	471.3471	−1.88	Maslinic acid	C30H47O4	423,405,393,249	−	+	[61]	Pentacyclic triterpenes
18.10	299.0586	8.32	Kaempferide	C16H12O6	284,227	+	−	[39]	Flavonoids
19.33	277.2170	−1.08	Linolenic acid (C18:3)	C18H30O2	---	+	−	[66]	Fatty acids
20.75	301.2171	−0.68	Methenolone	C20H30O2	220,205	+	−	[77]	Steroids
22.16	455.3522	−1.91	Betulinic acid	C30H48O3	---	−	+	[61]	Pentacyclic triterpenes
23.09	255.2329	0.00	Palmitic acid (C16:1)	C16H32O2	---	+	+	[39]	Fatty acids
23.81	281.2485	−0.37	Oleic acid (C18:1)	C18H34O2	---	+	+	[66]	Fatty acids

+ = present and − = absent.

**Table 3:** The effects of *E. arvense* and *O. europaea* extract administration on sperm characteristics in MTZ-treated rats.

Groups	Viability%	Abnormalities%	Concentrations (×10^6^/mL)	Motility%
GI (control)	75 ± 2.1 ^b^	10 ± 0.8 ^b^	29 ± 0.8 ^b^	76 ± 0.8 ^b^
GII (MTZ)	37 ± 1.1 ^a^	40 ± 2.1 ^a^	9.1 ± 1.1 ^a^	29 ± 1.8 ^a^
GIII (EA)	75 ± 0.8 ^b^	9 ± 0.5 ^b^	27.4 ± 1.2 ^b^	74 ± 0.9 ^b^
GIV (OE)	75 ± 0.5 ^b^	12.6 ± 1.7 ^b^	28.6 ± 0.8 ^b^	75 ± 2.8 ^b^
GV (MTZ + EA)	57 ± 2.1 ^ab^	22.6 ± 1.1 ^ab^	28.2 ± 1.1 ^b^	75± 1.5 ^b^
GVI (MTZ + OE)	42 ± 1.4 ^ab^	28 ± 1.5 ^ab^	21.6 ± 1.2 ^ab^	50 ± 1.8 ^ab^

Data are presented as mean ± SE (*n* = 8). Different letters in the same column indicate statistical significance at *p* < 0.05.

## Data Availability

All data are available from the corresponding author upon reasonable request, due to institutional policy and ethical constraints.
